# Public Awareness Regarding the Differences Between Ophthalmologists and Optometrists Among Adults Living in Makkah Province, Saudi Arabia

**DOI:** 10.7759/cureus.68739

**Published:** 2024-09-05

**Authors:** Abdulrahman M Almatrafi, Nawaf Almarzouki, Marshad A Almutairi, Ahmad M Ghandorah, Badran S Alqurashi, Rawan A Tash, Ibrahim S Aladni

**Affiliations:** 1 Medicine, King Abdulaziz University Faculty of Medicine, Jeddah, SAU; 2 Ophthalmology, King Abdulaziz University Hospital, Jeddah, SAU; 3 Medicine, King Abdulaziz University Hospital, Jeddah, SAU; 4 General Physician, King Abdulaziz University Hospital, Jeddah, SAU

**Keywords:** general population, ophthalmologist, optometrist, public awareness, saudi - non saudi

## Abstract

Background

Patients' knowledge of the differences between ophthalmologists and optometrists has been identified as a crucial factor influencing the choice of eye care services. This study aimed to assess the level of understanding of the differences between ophthalmologists and optometrists among the population in the Makkah province.

Methods

This cross-sectional study utilized an online questionnaire distributed to adults via social media platforms in the Makkah province, Saudi Arabia, from January to April 2023. The total number of respondents was 1,404.

Results

This study revealed that 464 (33.3%), 690 (49.5%), and 241 (17.3%) of the participants demonstrated a low, fair, and good understanding, respectively of the distinction between an optometrist and an ophthalmologist. Overall, 936 (66.7%) exhibited good knowledge.

The high level of knowledge is directly correlated with a history of previous eye examinations and the use of eyeglasses. The variables of age (specifically the 18-30-year cohort) and higher educational attainment (at or above university level) emerged as statistically independent predictors of sufficient knowledge acquisition. The odds ratios for younger age and a higher educational level were (confidence interval of 95%) 1.45 (1.11-1.88) and 1.42 (1.19-1.68), respectively.

Conclusion

This study revealed a fair public knowledge of the distinctions between optometrists and ophthalmologists. Additionally, we recommend the Ministry of Health encourage and sustain ongoing initiatives to enhance public awareness.

## Introduction

Eye care is one of the essential healthcare services that any healthcare organization should provide [1]. The World Health Organization's (WHO) call for international efforts to prevent avoidable blindness and visual impairment, coupled with the global expansion of optometry, has prompted a response from the World Optometry Council [1].

The Vision 2020 initiative, as outlined by the International Agency for the Prevention of Blindness (IAPB), was introduced to simplify the planning and development of national eye care programs. These programs aim to support comprehensive eye care services, incorporating integrated eye care professionals collaborating to provide preventive, curative, and rehabilitation services [2,3].

Optometrists and ophthalmologists are typically responsible for eye care, with their responsibilities varying based on the respective country's laws [4]. The patients' effectiveness in utilizing eye care services is closely tied to their awareness and understanding of the hierarchy of training, professional roles, capacities, and duties within the field [5,6]. There are many crucial distinctions between ophthalmology and optometry. Ophthalmologists undergo medical school training followed by a four-year ophthalmology training program to become specialists. Additionally, ophthalmologists develop subspecialties in medical or surgical eye care through fellowship programs in areas like retina, glaucoma, cornea, oculoplastic surgery, etc. These programs last one to two years. This extensive training spans 11-12 years, enabling ophthalmologists to perform eye procedures, diagnose and treat eye conditions, and prescribe medications [7,8]. On the other hand, optometrists, serving as primary healthcare providers for the eyes and visual system, offer comprehensive eye and vision care. Their scope includes refraction, prescription, detection, diagnosis, treatment of eye diseases, and rehabilitation for vision-related illnesses. The duration of specialization in optometry varies by country, taking approximately four to five years for optometry specialists to complete their education, including graduation from an optometry college and a mandatory internship year [9].

Optometrists and ophthalmologists can perform procedures and prescribe specific eye medications [10]. However, the scope of optometry services varies by country [4]. Notably, in Saudi Arabia, optometrists have different authority from physicians; instead, they may refer patients to ophthalmologists for treatments beyond their expertise [6]. On the other hand, ophthalmologists are typically responsible for prescribing various medications and conducting various surgical procedures [11]. It is crucial for individuals receiving eye care to be aware of the essential distinctions between these two specialists because their awareness has been linked to the healing process of injured eyes. Being informed can mitigate potential delays that may affect eye morbidity. Moreover, educating patients about eye care professionals can reduce therapy costs and save time [12-17].

A study conducted in 2016 in Nigeria revealed that awareness of the distinctions between the two specialties was positively correlated with educational status and prior experience with eye exams [17]. An Etawah, India, study conducted in 2019 on public knowledge of optometry found that 61.2% of participants needed to be educated about the role of optometrists in eye healthcare, and that 59.6% of respondents were unaware of the differences between optometrists and ophthalmologists [18].

The public's awareness of the two disciplines was evaluated in a 2018 study carried out in Riyadh, Saudi Arabia. According to the findings, only 789 (50%) of the participants had appropriate knowledge, with 505 (32%) having inadequate knowledge and 285 (18%) exhibiting excellent knowledge [8]. Furthermore, people's knowledge was found to be significantly impacted by age and educational background in the Riyadh study [8]. 

Owing to the need for further studies on this aspect in the Makkah province of Saudi Arabia, our objective was to assess the public awareness and knowledge among adults in the Makkah region regarding the distinctions between ophthalmologists and optometrists.

## Materials and methods

Study design, location, and time

A cross-sectional study was performed in the Makkah province, Saudi Arabia, from January to April 2023.

Ethical considerations

The study received ethical approval from the Research Ethics Committee at King Abdul-Aziz University Hospital in Jeddah, Saudi Arabia (HA-02-J-008).

Study participants

The study focused on adults aged 18 and above residing in the Makkah province, encompassing Saudi and non-Saudi populations.

Sample size

 According to the Saudi General Authority for Statistics, the population of the Makkah province, comprising both Saudis and non-Saudis, was reported as 9,033,491 individuals in 2019 [19]. A RAOSOFT web page (Raosoft, Inc., Seattle, WA) was used to estimate the sample for this study with a margin of error of 3% and a confidence interval (CI) of 97% [20]. The minimal acceptable sample size was 1,308 responses (20). For analysis, a total of 1,395 responses were included in this study.

Data collection

A Google Form was used for data collection, based on a validated questionnaire from a 1994 study in Los Angeles that assessed the public knowledge of ophthalmologists and optometrists [12]. The questionnaire was shared through social media platforms like X (formerly Twitter) and distributed in public places such as restaurants and coffee shops. To ensure relevance, we only included responses from individuals within our region, excluding those from outside. Randomization was achieved by targeting a broad audience through these methods, ensuring a diverse and geographically representative sample. The questionnaire was translated into Arabic by the institute's translation committee at King Abdulaziz University. The questionnaire was organized into three sections. The first section was dedicated to obtaining consent. The second section focused on collecting data on participants' previous eye examinations and demographic information such as age, gender, educational level, occupation, marital status, and nationality. The final section comprised 11 questions assessing participants' knowledge: seven questions focused on specific tasks, offering five possible answers (ophthalmologist, optometrist, both, none, or not sure), and four questions presented statements related to training requirements, with three response options (yes, no, or not sure).

Knowledge scores

The knowledge questions were adapted from a prior study conducted in Saudi Arabia (7). Poor knowledge was defined as an average score of <50%; fair knowledge was considered an average score between 50% and <75%; and good knowledge was identified with an average score of ≥75%. Scores ≥50% (fair and good knowledge scores) represented satisfactory knowledge.

Data analysis

The data were subjected to statistical analysis using the statistical software IBM SPSS version 22 (IBM Corp., Armonk, NY). Qualitative data, presented as numbers and percentages, were evaluated for associations between variables using the Chi-squared test (χ2). The Mann-Whitney U test was employed to evaluate associations for quantitative non-parametric variables expressed as mean and standard deviation (mean ± SD). Correlation analysis was performed using Spearman's test, and statistical significance was defined as a p-value <0.05.

## Results

This study aimed to evaluate the level of public knowledge and awareness among individuals in the Makkah province, Saudi Arabia regarding the differences between ophthalmologists and optometrists. Table 1 presents data from 1,395 participants enrolled in this study. Most participants (858; 61.5%) fell within the 18-30 age group, and 845 (60.6%) were male. Furthermore, 1,036 (74.3%) held a university degree or above, with 553 (39.6%) being students and 550 (39.4%) being employed. More than half (809: 58%) were single, and 819 (58.7%) had health insurance. Additionally, Table 1 shows that 1,112 (79.7%) of the participants had previously undergone an eye examination, 33.6% wore contact lenses, and 826 (59.2%) wore eyeglasses.

**Table 1:** Distribution of the participants based on their demographic information, previous eye examinations, and use of contact lenses or eyeglasses (N.: 1,395)

Variable	N (%)
Age	
18–30	858 (61.5)
31–50	393 (28.2)
51–65	144 (10.3)
Gender	
Female	550 (39.4)
Male	845 (60.6)
Nationality	
Saudi	1,288 (92.3)
Non-Saudi	107 (7.7)
Educational level	
Illiterate	3 (0.2)
Can read and write	2 (0.1)
Elementary	4 (0.3)
Intermediate	26 (1.9)
Diploma	5 (0.4)
High school	319 (22.9)
University degree and above	1,036 (74.3)
Occupation	
Private business	70 (5)
Student	553 (39.6)
Unemployed	143 (10.3)
Retired	80 (5.7)
Employed	549 (39.4)
Marital status	
Widowed	8 (0.6)
Single	809 (58)
Married	533 (38.2)
Divorced/separated	45 (3.2)
Possession of health Insurance	
No	819 (58.7)
Yes	576 (41.3)
Have you previously had an eye exam?	
No	283 (20.3)
Yes	1,112 (79.7)
Have you ever worn contact lenses?	
No	926 (66.4)
Yes	469 (33.6)
Have you ever worn eyeglasses?	
No	569 (40.8)
Yes	826 (59.2)

Table 2 depicts participants' knowledge regarding the differences between ophthalmologists and optometrists. Most participants 841 (60.3%) correctly stated that ophthalmologists undergo medical school training to become doctors. However, only 268 (20.5%) accurately stated that optometrists test vision, prescribe eyeglasses 601 (43.1%), fit contact lenses 787 (56.4%), and grind lenses 866 (62.1%). On the other hand, most participants 980 (70.3%) correctly identified that ophthalmologists perform cataract operations and use lasers to treat eye diseases 1,153 (82.7%).

**Table 2:** Participants' responses to knowledge questions regarding the differences between ophthalmologists and optometrists (N.: 1,395) * = correct answer

Variable	Both, N. (%)	None, N. (%)	Not sure, N. (%)	Ophthalmologist, N. (%)	Optometrist, N. (%)
Which specialist attended medical school as part of the training to become a doctor?	397 (28.5)	28 (2)	107 (7.7)	841 (60.3) *	22 (1.6)
Which specialist conducts vision tests?	553 (39.6)	11 (0.8)	89 (6.4)	456 (32.7)	286 (20.5) *
Which specialist prescribes eyeglasses?	396 (28.4)	14 (1)	86 (6.2)	298 (21.4)	601 (43.1) *
Which specialist fits contact lenses?	212 (15.2)	37 (2.7)	160 (11.5)	199 (14.3)	787 (56.4) *
Which specialist grinds lenses?	167 (12)	71 (5.1)	144 (10.3)	147 (10.5)	866 (62.1) *
Which specialist performs cataract surgeries?	95 (6.8)	20 (1.4)	121 (8.7)	980 (70.3) *	179 (12.8)
Which specialist uses lasers to treat eye diseases?	76 (5.4)	16 (1.1)	61 (4.4)	1153 (82.7) *	89 (6.4)

Table 3 shows that 1,102 (79%) of the participants correctly stated that ophthalmologists are authorized to test eyes for glaucoma and treat glaucoma with medications 985 (70.6%). In contrast, 256 (19%) correctly identified that optometrists can test eyes for glaucoma but are not authorized to treat it with medications 801 (57.4%).

**Table 3:** Participants' responses to knowledge questions regarding the differences between ophthalmologists and optometrists (N.: 1,395) * = correct answer

Variable	No, N. (%)	Not sure, N. (%)	Yes, N. (%)
Ophthalmologists have the authority to perform glaucoma tests on the eyes.	42 (3)	251 (18)	1,102 (79)*
Optometrists have the authority to perform glaucoma tests on the eyes.	672 (48.2)	458 (32.8)	265 (19) *
Ophthalmologists have the authority to treat glaucoma with medications.	99 (7.1)	311 (22.3)	985 (70.6)*
Optometrists have the authority to treat glaucoma with medications.	801 (57.4) *	391 (28)	203 (14.6)

**Figure 1:**
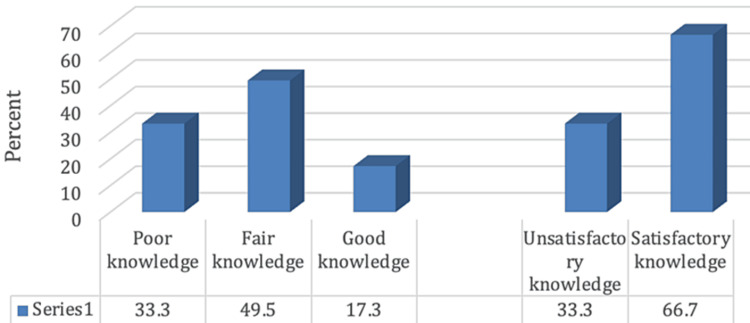
Percentage distribution of the participants according to knowledge level regarding the difference between ophthalmologists and optometrists

**Figure 2:**
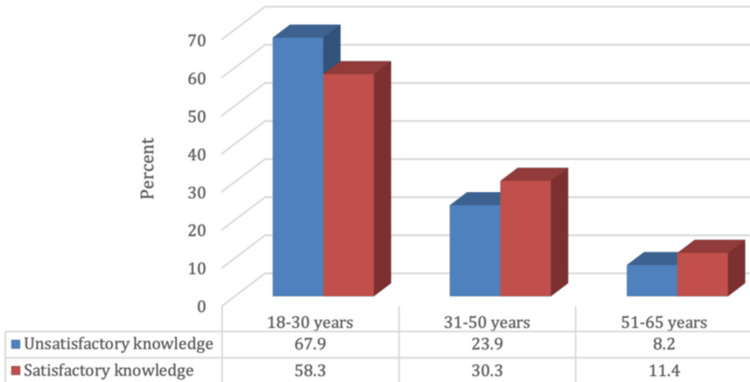
Relationship between participants' knowledge level regarding the differences between ophthalmologists and optometrists and their age (N.: 1,395) χ2= 12.12, p-value = 0.002

**Figure 3:**
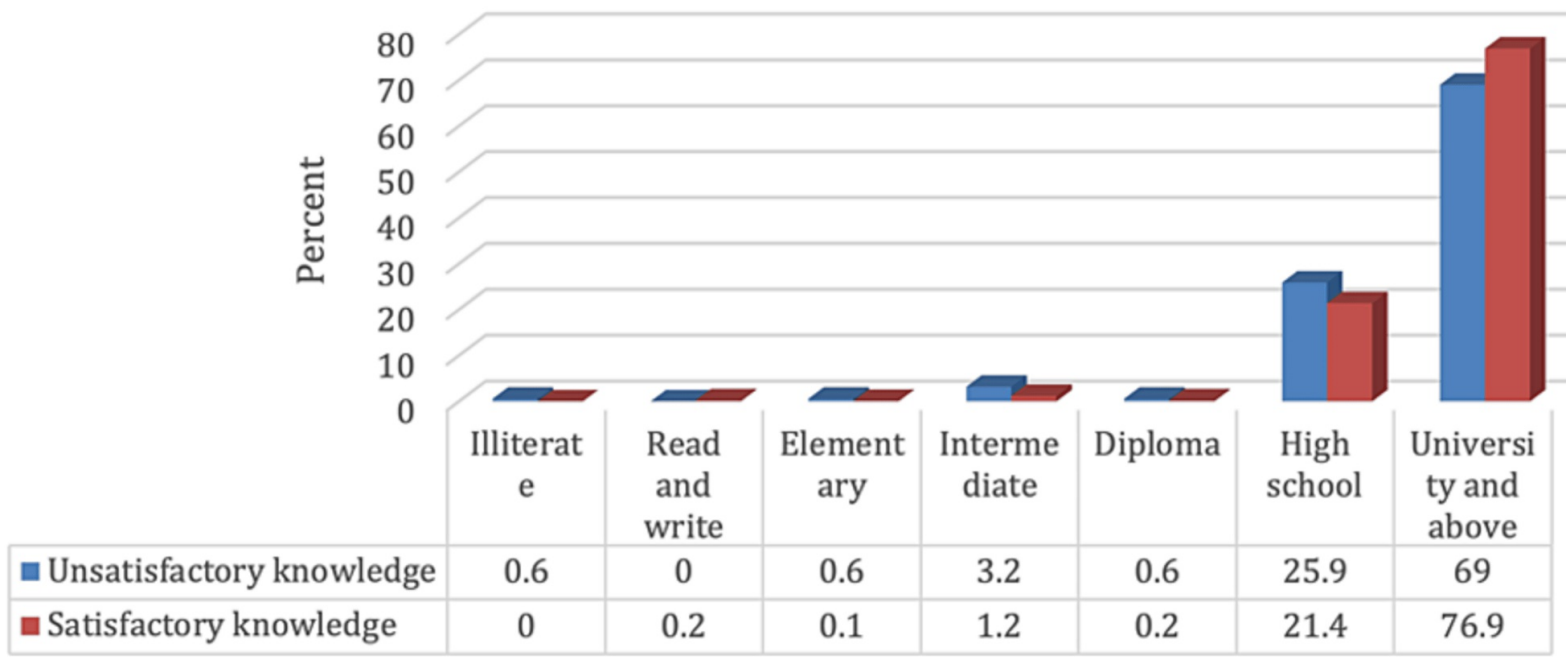
Relationship between participants' knowledge level regarding the differences between ophthalmologists and optometrists and their educational level (N.: 1,395)

Figure 1 delineates that 464 (33.3%), 690 (49.5%), and 241 (17.3%) of participants demonstrated poor, fair, and good knowledge regarding the disparities between ophthalmologists and optometrists, respectively, with 936 (66.7%) demonstrating satisfactory knowledge. Furthermore, satisfactory knowledge concerning the distinctions between ophthalmologists and optometrists was significantly higher among participants in the younger age group (18-30 years) and those with a higher educational level (university degree and above), as portrayed in Figures 2, 3 (p-values 0.002 and <0.001), respectively.

Table 4 demonstrates that participants who were employed and students had considerably greater levels of satisfactory knowledge regarding the distinctions between optometrists and ophthalmologists (p-value <0.05). Additionally, satisfactory knowledge was significantly higher among participants with previous eye exams and those wearing eyeglasses (p-value <0.05).

**Table 4:** Relationship between participants' demographic information, previous eye exams, wearing contact lenses, wearing eyeglasses, and their knowledge level regarding the differences between ophthalmologists and optometrists (N.: 1,395)

Variable	Unsatisfactory knowledge, N. (%)	Satisfactory knowledge, N. (%)	χ^2^	P-value
Gender			0.12	0.722
Female	186 (40.1)	364 (39.1)		
Male	278 (59.9)	567 (60.9)		
Nationality			0.09	0.763
Saudi	427 (92)	861 (92.5)		
Non-Saudi	37 (8)	70 (7.5)		
Occupation			15.42	0.004
Private business	37 (8)	33 (3.5)		
Student	180 (38.8)	373 (40.1)		
Unemployed	51 (11)	92 (9.9)		
Retired	20 (4.3)	60 (6.4)		
Employed	176 (37.9)	373 (40.1)		
Marital status			1.74	0.187
Single	298 (64.2)	564 (60.6)		
Married	166 (65.8)	367 (39.4)		
Possession of health insurance			2.39	0.122
No	259 (55.8)	560 (60.2)		
Yes	205 (44.2)	371 (39.8)		
Have you previously had an eye exam?			13.36	<0.001
No	120 (25.9)	163 (17.5)		
Yes	344 (74.1)	768 (82.5)		
Have you ever worn contact lenses?			0.36	0.548
No	313 (67.5)	613 (65.8)		
Yes	151 (32.5)	318 (34.2)		
Have you ever worn eyeglasses?			13.47	<0.001
No	221 (47.6)	348 (37.4)		
Yes	243 (52.4)	583 (62.6)		

**Figure 4:**
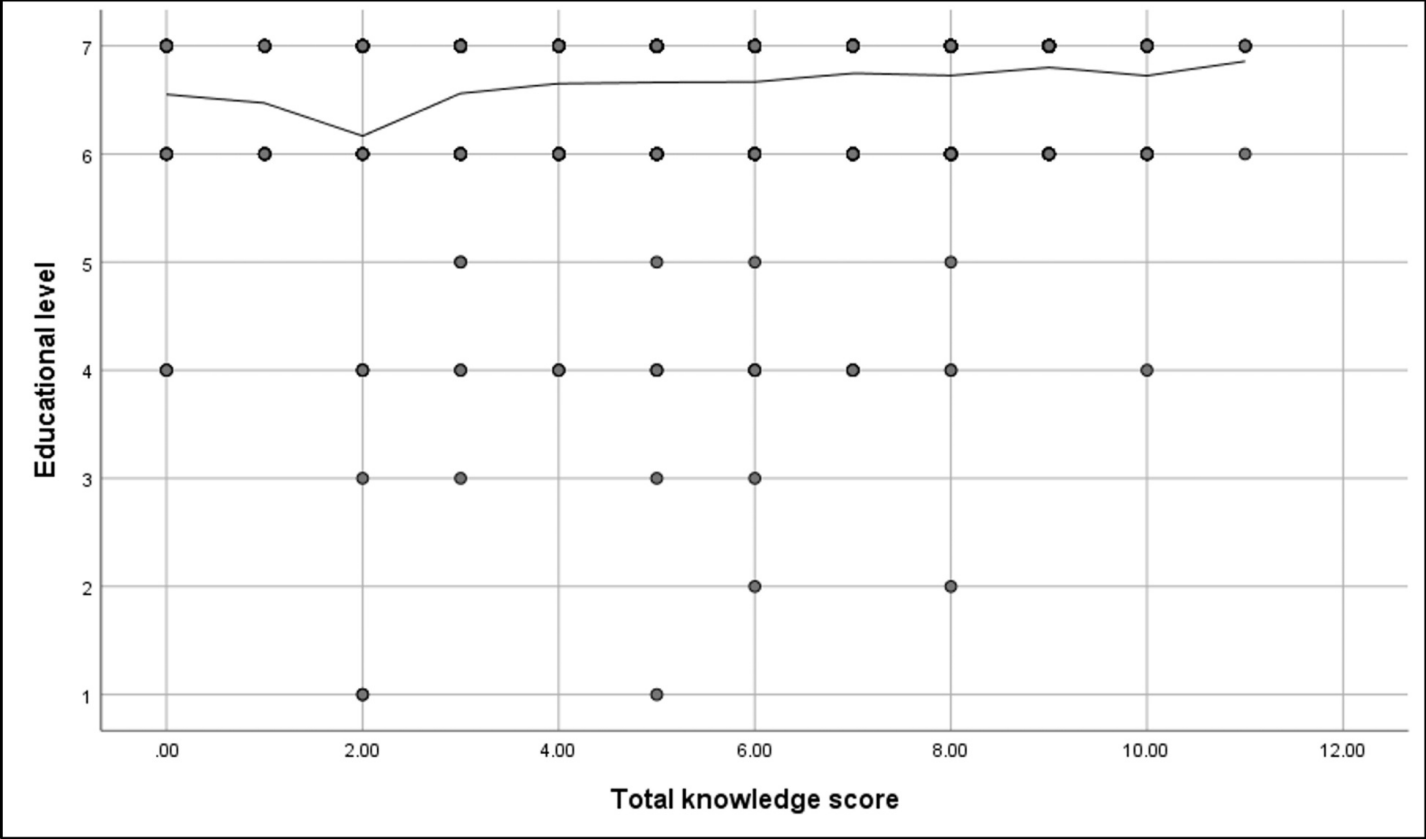
Spearman's correlation analysis between participants' knowledge score and their educational level (N.: 1,395) r= 0.1, p-value = <0.001

**Figure 5:**
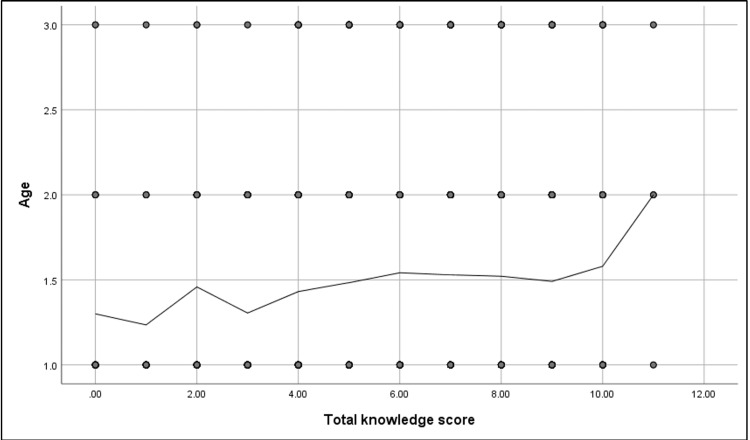
Spearman's correlation analysis between participants' knowledge score and their age (N.: 1,395) r= 0.09, p-value = 0.001

Figures 4, 5 show that a significant positive correlation was found between the participants' knowledge score and their educational level (r= 0.1, p-value = <0.001) and age (r= 0.09, p-value = 0.001).

## Discussion

The overlap in roles between ophthalmologists and optometrists has led to considerable public confusion over their respective responsibilities in the eye healthcare system. This study is the first to evaluate the awareness of the adult population in the Makkah province regarding the distinctions between ophthalmologists and optometrists and the second such study in Saudi Arabia after the survey was conducted in the Riyadh Region by Adebisi et al. [8]. Previous studies have examined the general population's ability to differentiate between ophthalmologists and optometrists, spanning countries such as the United States, Nigeria, India, and Saudi Arabia [8,12,17,21-24].

In our study, 936 (66.7%) of the participants demonstrated satisfactory general knowledge regarding the differences between ophthalmologists and optometrists. Comparatively, a study in Nigeria found that 219 (55.6%) of the participants were aware of the differences in training requirements, and 18.5%-74.9% were aware of the specific tasks of both specialists [17]. Additionally, a study in the United States revealed that 55.6% of survey questions were answered correctly [24]. Our study suggests a higher knowledge level compared to previous studies, likely due to the influence of social media platforms in enhancing public awareness. However, the study conducted in Riyadh reported even higher results, with 68% of participants demonstrating good knowledge [8]. This variation in prevalence may be attributed to several factors, including differences in sample demographics, the effectiveness of educational campaigns in the region, and varying levels of access to healthcare information. Such factors could contribute to the observed discrepancies in knowledge levels among different populations.

Our study shows that educational level was a significant demographic factor influencing satisfactory knowledge levels, consistent with previous research that identified a correlation between education and knowledge levels [23-26]. Additionally, occupation, particularly being a student (university degree and above), significantly impacted knowledge levels (p-value <0.05), supporting similar findings from other studies [8,24,27].

Furthermore, our results indicated that the younger age group demonstrated a significant association with good knowledge, which contrasts with some previous findings. This discrepancy may be attributed to the composition of our sample, which predominantly consisted of young adults aged 18-27. In contrast, a survey conducted in Nigeria found no significant relationship between age and knowledge levels [17]. The variation in these findings could be explained by differences in educational systems, cultural contexts, and access to information across the studied populations.

Regarding the influence of eye examinations on knowledge, participants who had undergone an eye examination demonstrated greater knowledge (79.7%) compared to those who had not (20.3%). This finding suggests that personal experiences with eye examinations may enhance understanding of eye health and related topics, aligning with previous studies [8,17]. However, it is important to note that the educational background of individuals may also play a significant role in shaping their knowledge about eye examinations. Therefore, future research should explore how education impacts individuals' understanding and attitudes toward eye health.

Wearing eyeglasses was positively associated with acceptable knowledge, consistent with studies by Aldebasi et al. and Wilson et al. [8,12] but contrasting with the findings of Eze et al., where those who did not wear eyeglasses had higher knowledge levels [17]. Not wearing contact lenses was associated with a higher knowledge level in our study, aligning with the results of Eze et al. [17].

While 931 (66.7%) of the participants demonstrated satisfactory knowledge, the remaining 464 (33.3%) required additional information to distinguish the roles of ophthalmologists and optometrists in the eye care health system. These findings emphasize the need for awareness campaigns to educate the public on these differences, aiding in informed decision-making and financial management within the eye healthcare system.

Our findings demonstrate that two variables independently predicted satisfactory knowledge levels among the participants. The first variable, age, showed a significant positive association with satisfactory knowledge. This implies that individuals in the younger age group (18-30 years) are more likely to have better knowledge regarding the differences between ophthalmologists and optometrists. This suggests that individuals within this age range may be currently enrolled in or have recently completed higher education, potentially giving them access to educational resources, including information related to healthcare.

The other significant predictor was educational level, demonstrating that higher levels of education were associated with better knowledge. This finding aligns with previous studies and may be explained by individuals with higher education levels having better access to educational resources such as books, academic journals, and online databases. This increased access to information may give them more opportunities to learn about healthcare topics, including the differences between ophthalmologists and optometrists.

However, other variables, including gender, nationality, occupation, marital status, possession of health insurance, previous eye exams, wearing contact lenses, and wearing eyeglasses, did not exhibit significant associations with satisfactory knowledge. In the context of our study, these variables may play a minor role in determining participants' awareness of the differences between ophthalmologists and optometrists. Improving knowledge in these areas could lead to better health-seeking behaviors and more informed decisions regarding eye care. Enhancing awareness among diverse demographic groups may ultimately contribute to increased utilization of appropriate eye care services and better overall health outcomes.

The study has several limitations. First, the sample may not represent the awareness of the entire Saudi and non-Saudi population. Second, distributing our survey to the entire population was challenging because there was no official platform to publish our questionnaire.

## Conclusions

This study evaluated the general public's awareness of the distinctions between optometrists and ophthalmologists, concluding that the community's knowledge level is fair. However, there is room for improvement, and virtual campaigns through social media, initiated by the Ministry of Health and policymakers, could play a crucial role in enhancing public awareness and facilitating informed decisions regarding eye healthcare. We recommend that future researchers increase the sample size to encompass all regions of Saudi Arabia.

## Appendices

Questionnaire Public awareness regarding the difference between ophthalmologists and optometrists among adults living in Makkah province, Saudi Arabia Age and Gender 1.      Age o   18-30 o   31-50 o   51-65 o   >65 2.      Gender o   Male o   Female Socio-economic characteristics 1.      Educational level o   Elementary o   Intermediate o   High school o   Higher education  2.      Occupation o   Private business o   Employed o   Retired o   Student o   Unemployed 3.      Marital status o   Single o   Married o   Divorced/separated o   Widowed 4.      Possession of health insurance o   Yes o   No 5.      Nationality o   Saudi o   Non-Saudi Medical status 1.      Have you previously had an eye exam? o   Yes o   NO 2.      Have you ever worn contact lens/spectacles? o   Yes o   NO knowledge questions and statements Section A: questions 1.      Which of them went to medical school to become a doctor as part of their training? ophthalmologist 2.      Which of them tests your vision? Both 3.      Which of them prescribes glasses? Both 4.      Which of them fits contact lenses? Both 5.      Which of them grinds lenses? None 6.      Which of them performs cataract operations? ophthalmologist 7.      Which of them uses lasers to treat eye diseases? ophthalmologist Answers (ophthalmologist, optometrist, both, none, or not sure) Section B: Statements 1.      An ophthalmologist is allowed to test the eyes for glaucoma. yes 2.      An optometrist is allowed to test the eyes for glaucoma. yes 3.      An ophthalmologist is allowed to treat glaucoma with medications. yes 4.      An optometrist is allowed to treat glaucoma with medications. no Answers (yes, no, or not sure).
